# Impact of two different patient decision aids in prosthodontic consultations: a prospective randomized controlled study

**DOI:** 10.1007/s00784-023-05375-7

**Published:** 2023-11-27

**Authors:** Silvia Brandt, Hans-Christoph Lauer, Jan-Frederik Güth, Sarah Bühling, Babak Sayahpour, Georgios Romanos, Anna Winter

**Affiliations:** 1 Department of Prosthodontics, Center for Dentistry and Oral Medicine (Carolinum), Goethe University Frankfurt, Theodor-Stern-Kai 7, Building 29, 60596 Frankfurt am Main, Germany; 2 Department of Periodontics and Endodontics, Stony Brook School of Dental Medicine, New York, NY USA; 3 Department of Prosthodontics, University Hospital Würzburg, Würzburg, Germany

**Keywords:** Shared decision-making, Patient decision aid, Prosthodontic consultation, Treatment planning, Patient satisfaction

## Abstract

**Objectives:**

Different approaches to prosthodontic consultation, all involving a strong focus on shared decision-making, were analyzed from the perspective of patients by inter-group comparisons. No patient decision aid (PDA) was used in the control group, a paper-based PDA in test group 1, and a software-based PDA in test group 2.

**Materials and methods:**

Seventy-five patients were prospectively randomized to the control group or a test group. All patients then rated the consultation on a questionnaire, six key items of which were analyzed, along with the time spent on each consultation.

**Results:**

Overall satisfaction was highest in test group 2, with a significant difference from the control group (*p* = 0.015). Test group 2 showed the most favorable ratings for all six questionnaire items, which invariably was significant compared to the control group (*p* = 0.032). Test group 1 significantly differed from test group 2 based on two items (consultation was adequately intelligible: *p* = 0.011; consultation was adequately comprehensive: *p* = 0.034) but not from the control group based on any item (*p* = 0.070).

**Conclusions:**

Within the limitations of this study, the use of a software-based PDA, in particular, can be recommended based on patient satisfaction and was associated with the shortest sessions for consultation.

**Clinical relevance:**

Patients are routinely faced with a wealth of information in dental offices and may be overwhelmed especially by prosthetic treatment options and decision requirements. Our findings shed some light on the nature of aids that may truly be helpful in the process of shared decision-making.

**Trial registration:**

ClinicalTrials.gov.Identifier: ISRCTN11472465.

## Introduction

Dental as well as medical therapies are increasingly provided with a focus on individual patient requirements and emotional well-being. Any patient-centered care of this type needs to be firmly grounded in a meaningful relationship between the patient and the dental team, communication being the key to establishing such a relationship [1]. It takes communicative and interpersonal skills on the clinician’s part to gather all the patient-specific information that is required for a correct diagnosis and to select an appropriate process that will optimally meet each individual’s therapeutic needs [2].

Patient outcomes can be improved further by one-on-one consultations in accordance with the principle of “shared decision-making” prior to treatment [3, 4]. The aim of this process is to create a trustful relationship that will allow clinicians to pinpoint individual needs and raise their patients’ knowledge of underlying pathologies to a level enabling the patients to make informed decisions related to their own health [4, 5]. In this process, patients are supplied with evidence-based information on available treatment options, pointing out the specific benefits and harms of each option, exploring their compatibility with personal preferences, and discussing these points with the entire team [4, 6].

An evidence-based strategy of this type can ensure that the clinician’s and patient’s expectations will converge, thus avoiding discrepancies [6]. Patients involved in the deliberations will gain a better understanding of their diagnoses, treatment plans, and medications to be used. Moreover, such patient involvement for shared decision-making is capable of improving clinical outcomes, minimizing risks, and enhancing patient autonomy, in addition to protecting fundamental rights to information and freedom of choice [7, 8].

Shared decision-making is, by no means, a new concept of medical ethics [9] and continues to be widely discussed in many fields of medicine [10–13]. Definitions are not uniform but do share some components [10]. The reference point we use for the present report is the four steps of communication presented by Stiggelbout et al. [11]. In short, this process requires clinicians to engage in a dialog with their patients (i) informing them that a decision is to be made and that their opinion is important; (ii) explaining treatment options and their pros and cons; (iii) discussing the patients’ preferences and supporting them in deliberation; and (iv) discussing whether they prefer to make their own decision or leave it up to the clinician.

In clinical practice, these standards are often not fully met [11], and this concern was voiced in a general medical context where shared decision-making is more commonly discussed in life-or-death matters. Failure to meet these standards may be even more prevalent in dental offices where their clinical relevance is more likely confined to patients being routinely faced with a variety of decision requirements. One-on-one consultations for prosthetic treatment, in particular, will commonly include a wealth of information and treatment options to be discussed, which poses particular challenges with regard to ensuring autonomous decisions and informed preferences. For this reason, there is an everyday need for shared decision-making in dentistry [12].

Patient decision aids (PDAs) are tools that can be used to support the task of shared decision-making [13]. They are believed to increase the effectiveness of communication between clinicians and patients, thus optimizing patient information and patient care [14, 15], and are designed to inform about clinical problems and outcome probabilities in an easy-to-grasp fashion. Typically, they contain examples for illustration, in an effort to make sure that treatment decisions are consistent with personal values [15]. That said, while PDAs are commonly used to support conversations for prosthodontic consultation, this application is not well investigated. To the authors’ knowledge, no comparative studies are available on the difference they actually make in this context.

Against this background, we designed a patient-centered survey to investigate the quality of prosthodontic consultations involving or not involving two commonly used PDAs. As null hypotheses, it was assumed that statistical differences in patient ratings would be observable neither (i) for both PDAs versus shared decision-making by oral conversation only; nor (ii) between consultations supported by either one of both PDAs.

## Materials and methods

### Preparations and recruitment

The survey was designed as a prospective study and was conducted at the Department of Prosthodontics (Center for Dentistry and Oral Medicine, Frankfurt, Germany) over a 1-year period from January 2019 to January 2020. Having obtained approval from the institutional ethics committee (ref. 71/14), potential participants were recruited from patients in need of dental prosthetic treatment who had an appointment for treatment planning. Only patients were included who were ≥ 20 years old, had an adequate command of spoken and written German, gave their consent to participating in the study, and for whom recent (≤ 6 months) radiographic documentation was available. Cases not found to require prosthodontic treatment, pregnant women, as well as patients with a record of drug or alcohol abuse or with known allergies to dental materials, were excluded.

### Randomization and consultations

Seventy-five patients were thus included and randomly assigned to three equal-sized groups of 25 patients, each defined by a slightly different approach to prosthodontic consultation. All three approaches had in common that the patient went through a dialog with a defined clinician in a defined room based on the findings of a clinical intraoral examination and on the radiographic records (see above).

**Fig. 1:**
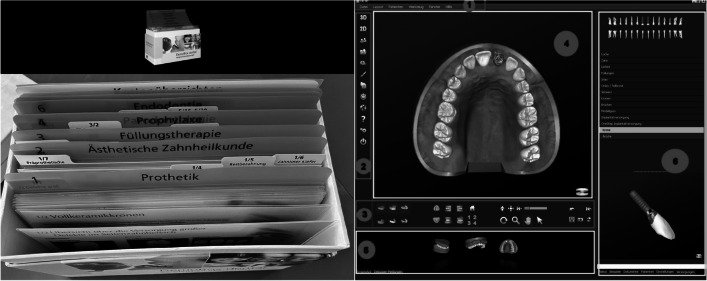
Paper-based PDA (left) and software-based PDA (right)

All conversations were conducted by the same clinician in the same room. As particularly short or long clinical experience was not considered desirable, a clinician with a medium experience level of 5 years was selected. No calibration took place, but group bias by learning effects was avoided by cycling through the three groups in the same order. Care was taken in the conversations to discuss the pros and cons of different treatment options, placing a strong focus on the patient’s own views to arrive at treatment decisions meeting the criteria of “shared decision-making.” More specifically, the same four steps of communication were followed as outlined in the Introduction [11]. As described in the paragraphs that follow, the conversations in the three groups differed by whether or not they involved either one of two commonly used PDAs (Fig. 1).

- Control group (no PDA). In this group, the clinician did not use any aids other than the existing radiographs and the intraoral findings on a sheet of paper in explaining treatment options to the patients for shared decision-making.
- Test group 1 (paper-based PDA). In this group, the clinician adopted the same approach as in the control group but additionally used a loose-leaf publication for patient information that comes in a cardboard box (DemoBox dental; Spitta, Balingen, Germany). Hence, the alternative options were both explained orally and demonstrated to the patient via paper-based illustrations of conservative, periodontal, and prosthodontic treatment. With this PDA, selective information could be provided depending on the number of missing teeth (single spaces, multiple spaces, or edentulism in anterior or posterior segments) for different prostheses (fixed, removable, or combined) and abutments (teeth or implants). Pros and cons of the alternative options could also be illustrated using this PDA.
- Test group 2 (software-based PDA). In this group, the clinician adopted the same approach as in the control group but additionally used a software-based library for patient information (Dental Explorer 3D; Quintessence Publishing, Berlin, Germany) to support the conversation. In this way, specific aspects of the case were visualized on a computer screen. This PDA includes not only images but also three-dimensional models and video sequences illustrating a variety of prosthetic designs. It can also be used to create digital casts for the case-specific situation to be visualized, so that alternative options of treatment could be demonstrated and their expected outcomes previewed together with the patient.

**Fig. 2:**
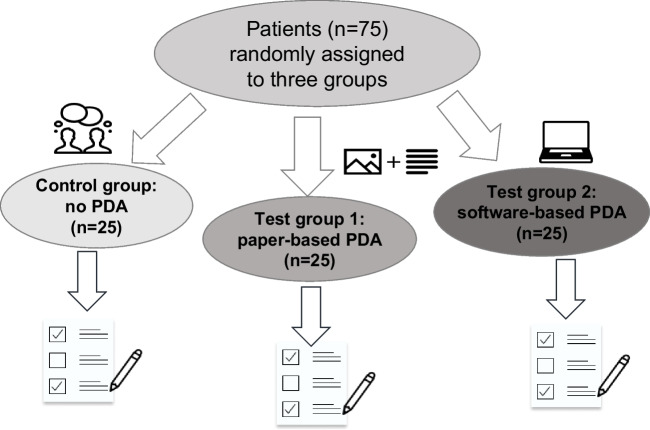
Schematic roadmap to the study

A schematic roadmap to the study is provided in Fig. 2. Both PDAs in the test groups were state of the art during the study, are easily affordable for any dental practice, and can be readily learned and used by any practitioner. The software-based PDA can be acquired (e.g., on DVD-ROM) without incurring an overhead in subscription expenses and does not involve any special hardware requirements. The idea was not to explore artificial intelligence (an adequately usable format was not available at the time) but to compare the haptic feel of real paper versus animations on a computer screen.

### Questionnaire and ratings

After this session for prosthodontic consultation, each of the 75 patients was asked to complete a questionnaire, which had been tested in advance in a reference group of ten patients. Table 1 summarizes its core items, including general questions about age and gender, language comprehension, previous experience with prosthodontic treatment, and what decision had ultimately been reached regarding the prosthetic design.

**Table 1:** Questionnaire completed by all 75 patients immediately after the prosthodontic consultation

Name	Age	□ Female		□ Male				□ This is my first/native language						□ This is not my first/native language						
Understanding of language:						Nonexistent		□	□	□	□	□	□	□	□	□	□	Perfect		
• I have chosen this kind of prosthetic restoration:			□ Removable		□ Fixed	□ Combined	• I had one or several teeth replaced in the past:												□ Yes	□ No
On a ten-point scale ranging from “strongly disagree” to “strongly agree” (please check the appropriate box for each of the following items), I feel that…																				
• …the prosthetic consultation has been adequately intelligible						**Strongly disagree**		**□**	**□**	**□**	**□**	**□**	**□**	**□**	**□**	**□**	**□**	**Strongly agree**		
• …I have been comprehensively informed by the consultation						**Strongly disagree**		**□**	**□**	**□**	**□**	**□**	**□**	**□**	**□**	**□**	**□**	**Strongly agree**		
• …different restorative options for my situation have been adequately presented						**Strongly disagree**		**□**	**□**	**□**	**□**	**□**	**□**	**□**	**□**	**□**	**□**	**Strongly agree**		
• …the sequence of planned treatment steps has been adequately explained						**Strongly disagree**		**□**	**□**	**□**	**□**	**□**	**□**	**□**	**□**	**□**	**□**	**Strongly agree**		
• …additional aids (e.g. pictures, models/casts, etc.) would have been helpful						**Strongly disagree**		**□**	**□**	**□**	**□**	**□**	**□**	**□**	**□**	**□**	**□**	**Strongly agree**		
• …overall, I was satisfied with the prosthetic consultation						**Strongly disagree**		**□**	**□**	**□**	**□**	**□**	**□**	**□**	**□**	**□**	**□**	**Strongly agree**		

This representation of the questionnaire is not an identical reproduction; the original language was German

Furthermore, all patients were also asked to provide ratings for the quality of the prosthodontic consultation itself by checking one of ten boxes on a Likert scale—resulting in a score of 1 (“strongly disagree”) to 10 (“strongly agree”)—for each of the six items. Specifically, they were asked to what extent they felt that.

- the consultation had been adequately intelligible
- the consultation had been adequately comprehensive
- different treatment options had been adequately presented
- the sequence of treatment steps had been adequately explained
- additional explanations would have been helpful
- they were satisfied with the consultation overall

While higher scores indicated higher satisfaction with five of these items, the reverse was true with one item (“additional explanations would have been helpful”). Hence, to avoid confusion, scores will be described as more or less “favorable” in this text.

### Statistical analysis

Data were analyzed with statistical software (IBM SPSS Statistics 29 (SPSS Inc. an IBM Company, Chicago, IL) and checked for normal distribution using a Kolmogorov–Smirnov test. The three patient groups were then analyzed for statistical differences—to be considered significant at *p* ≤ 0.05—using a chi-square, Kruskal–Wallis, and Mann–Whitney *U* test. In addition, internal consistency was assessed using Cronbach’s alpha test.

## Results

Of the 75 included patients, 50 had ultimately opted for fixed prostheses, while 10 had opted for removable dentures and 15 for combined removable and fixed prostheses. As shown in Table 2, pertinent data for the entire sample (*n* = 75) with regard to age, sex, and gender, as well as amounts of time spent on the prosthodontic consultations, did not yield a significant difference between the three consultation groups (*p* = 0.472). The slightly lower time requirements in test group 2 (see Table 2), while not qualifying as a noteworthy finding in its own right, may still be regarded as a “bonus,” considering that PDA-assisted sessions were not per se expected to consume less time.

**Table 2:** Demographic overview of the study population and the three consultation groups

	Total	Control group(no PDA)	Test group 1 (paper-based PDA)	Test group 2 (software-based PDA)
Number of patients (n)	75	25	25	25
Mean age ± SD (years)	59.4 ± 14.5	57.4 ± 14.6	61.5 ± 12.8	59.2 ± 16.2
Female versus male sex and gender (n)	38/37	12/13	14/11	12/13
Mean consultation length ± SD (min)	36.8 ± 13.5	37.5 ± 14.8	37.9 ± 15.3	35.0 ± 10.3
Median consultation length (Q_1_;Q_3_) (min)		35 (25;45)	35 (37.5;45)	35 (25;45)

No significant differences were noted (*p* = 0.472). *PDA*, patient decision aid; Q_*1*_, lower quartile (25th percentile);* Q*_*3*_, upper quartile (75th percentile)

For the questionnaire completed by all patients after the prosthodontic consultation, Cronbach’s alpha was found to range between 0.81 and 0.96. The internal consistency of items may therefore be considered moderate to high in this questionnaire. The layout shown in Table 1 is not a precise reproduction of the original questionnaire; the original language was German.

The results for the six items are summarized in Table 3. Note that the highest mean ratings (9.76 ± 0.44) for overall satisfaction with the prosthodontic consultation were reached in test group 2 (software-based PDA). These ratings were significantly higher only compared to the ratings (9.12 ± 1.05) in the non-PDA control group (*p* = 0.015). Indeed, the mean ratings for all six items of interest were most favorable in test group 2 (software-based PDA) compared to the other two groups, which again involved a significant advantage only in comparison to the non-PDA control group (*p* = 0.032).

**Table 3:** Intergroup comparison of patient ratings for six key questions of interest

	Control group (no PDA)			Test group 1 (paper-based PDA)			Test group 2 (software-based PDA)		
	Mean ± SD	Median (Q_1_;Q_3_)		Mean ± SD	Median (Q_1_;Q_3_)		Mean ± SD	Median (Q_1_;Q_3_)	
Consultation was adequately intelligible	9.2 ± 1.4	10 (9;10)	A	9.5 ± 0.7	10 (9;10)	A	9.9 ± 0.3	10 (10;10)	B
Consultation was adequately comprehensive	9.2 ± 1.4	10 (9;10)	A	9.6 ± 0.6	10 (9;10)	A	9.9 ± 0.3	10 (10;10)	B
Different options were adequately presented	9.2 ± 1.2	10 (9;10)	A	9.7 ± 0.7	10 (10;10)	A,B	10 ± 0.2	10 (10;10)	B
Sequence of treatment was adequately explained	8.8 ± 1.6	10 (8;10)	A	9.6 ± 0.7	10 (9;10)	A,B	9.8 ± 0.7	10 (10;10)	B
Additional explanations would have been helpful	5.4 ± 3.8	4 (2;10)	A	3.6 ± 3.1	2 (1;6.5)	A,B	2.6 ± 3.0	1 (1;2.5)	B
Ratings for overall satisfaction	9.1 ± 1.1	9 (8;10)	A	9.6 ± 0.5	10 (9;10)	A,B	9.8 ± 0.4	10 (9.5;10)	B

*A/B*, any horizontally aligned fields not containing the same letter involve a significant inter-group difference (*p* ≤ 0.05); *PDA*, patient decision aid; *Q*_*1*_, lower quartile (25th percentile); *Q*_*3*_, upper quartile (75th percentile)

Significantly, more favorable ratings were obtained in test group 2 (software-based PDA) than in test group 1 (paper-based PDA) regarding the extent to which the consultation was experienced as intelligible (9.88 ± 0.33 versus 9.48 ± 0.65; *p* = 0.011) and comprehensive (9.92 ± 0.28 versus 9.64 ± 0.57; *p* = 0.034) by the patients. All six questionnaire items were not associated with any significantly different ratings between test group 1 (paper-based PDA) and the non-PDA control group (*p* = 0.070).

## Discussion

In this patient-centered study of prosthodontic consultations, two different PDAs were compared for overall patient satisfaction and its constituent quality criteria. Compared to a control group not involving the use of a PDA, a statistically significant impact was demonstrable in a group involving the additional use of a software-based PDA during the consultations. Also, the results in this group were more favorable, including some statistically significant differences, than the results in a group involving the use of a paper-based PDA. Both null hypotheses underlying the study were therefore rejected.

While the mean ratings were, again, consistently more favorable in this latter group (paper-based PDA) than in the control group (no PDA), none of these differences was found to be statistically significant. This relative closeness of intergroup findings is less surprising when considering that all consultations, including those in the control group, were carried on with a focus on meeting the criteria of “shared decision-making” [11, 16]. This adherence is also reflected by high ratings for overall patient satisfaction even in the control group, which is consistent with previous results from the fields of esthetic dentistry and orthodontics, demonstrating that patient satisfaction improved after the principles of “shared decision-making” had been implemented in consultations [17, 18].

Despite this aforementioned lack of statistical significance, it should be noted that the general trend of ratings for overall patient satisfaction was clearly more favorable with the paper-based PDA (test group 1) than without a PDA (control group). In particular, the ratings for “different options were adequately presented” and “sequence of treatment was adequately explained” came fairly close to the ratings in test group 2 involving a software-based PDA (see Table 3). Therefore, even the loose-leaf publication used in test group 1 may have contributed quite effectively to increasing the patients’ understanding of alternative treatment options and of the treatment plans that had eventually been decided upon, which is also consistent with previous findings [19].

In orthodontics, Marshman et al. [20] likewise demonstrated high levels of satisfaction with information conveyed via an analog PDA, which decreased decisional conflicts in choosing between treatments. That said, even the best illustrations and the clearest language cannot change the fact that some patients may have difficulty grasping such information from paper [19]. Scenarios like these might well account for the better ratings with the software-based than with the paper-based PDA in the present study, and this assumption is, indeed, corroborated in very specific way by significantly better ratings for the consultations being experienced as adequately intelligible.

Nevertheless, the feeling among patients that additional aids would have been helpful was reduced in both PDA groups, and significantly so in test group 2 (software-based PDA) compared to the control group. Hence an active desire for a PDA does seem to exist on the patients’ part. It should also be considered, however, that not all patients are equally keen to play an active role in shared decision-making. Motamedi-Azari et al. [21] reported a gender difference in this regard, with female patients being more likely to prefer an active role in conversations for shared decision-making. Be that as it may, the additional use of a PDA could especially suit the needs of those who are happier with a passive role during the conversation, given that some of the points that the PDA inherently conveys will no longer have to be discussed in detail.

The highest levels of overall satisfaction with the prosthodontic consultations in the present study were reported in test group 2 (software-based PDA), even though the advantage over test group 1 (paper-based PDA) was not found to be statistically significant. Of course, any PDA that is used to complement a consultation session needs to include information about the clinical problem at hand, available treatment options, treatment outcomes, and examples for demonstration [15, 19]. It stands to reason that material of this type should increase patient satisfaction, and the trend we observed in favor of the software-based PDA can be readily attributed to its superior capabilities. The possibility of creating a virtual model of the patient’s dental status and to directly visualize the existing treatment options can also be readily associated with the significantly better ratings for adequate intelligibility of the consultations in this group than in the group involving the use of a paper-based PDA.

Visualizing case-specific scenarios is a hallmark of advanced technologies, one example being the “digital smile design” process, which educates patients by allowing them to view their own situation [22]. While this process does not yield information beyond its dedicated purpose, PDAs are designed to support both patients in arriving at decisions and clinicians in getting their messages across. Therefore, another parameter that was measured in the present study was the time spent on each consultation. Compared to the control group (no PDA), these durations were found to be shorter in test group 2 (software-based PDA) but longer, albeit not significantly, in test group 1 (paper-based PDA), which is consistent with findings of a Cochrane review on PDAs in medicine [23].

Several limitations of the present study should be mentioned. Firstly, any knowledge gained by the patients through the use of either PDA may be surmised from their ratings but was not assessed by objective criteria. Conclusions to this effect, which could be drawn from knowledge levels noted after versus before the consultations, are beyond the scope of this report. Also, the study did not factor in prosthetic treatment requirements, states of prosthetic restoration, or histories of treatment, given that past experiences might affect the outcomes of shared decision-making in prosthodontics [24].

Lastly, the questionnaire used in the study was not an established questionnaire, and its test–retest reliability was not investigated. Its content was, however, highly intelligible to all patients, and the authors are not aware of any other existing questionnaire that could have been harnessed or adapted to assess the usefulness of PDAs in prosthodontics. Also, the questionnaire had been pre-tested for clinical usability in a reference group of ten patients.

## Conclusions

Within the limitations of this study, the following conclusions can be drawn:

I. Both a paper-based and a software-based PDA turned out to be a useful addition to shared decision-making during prosthodontic consultations in the present study. Only the software-based PDA was found to improve both patient satisfaction and the perceived lucidity of explanations received in a statistically significant fashion.
II. While the use of either a paper-based or a software-based PDA can be recommended for prosthodontic consultations, it is suggested that better results and time savings may be attainable with an appropriate software-based solution.

## References

[1] Sharp M, Williams N, Tackett S, Hanyok LA, Christmas C, Rand CS, Ziegelstein RC, Record JD (2022). Observation tool to measure patient-centered behaviors on rounds in an academic medical center. Med Educ Online.

[2] Ha JF, Longnecker N (2010). Doctor-patient communication: a review. Ochsner J.

[3] Makaryus AN, Friedman EA (2005). Patient’s understanding of their treatment plans and diagnosis at discharge. Mayo Clin Proc.

[4] Hoffmann T, Bakhit M, Michaleff Z (2022). Shared decision making and physical therapy: what, when, how, and why?. Braz J Phys Ther.

[5] Redley B, McTier L, Botti M, Hutchinson A, Newnham H, Campbell D, Bucknall T (2019). Patient participation in inpatient ward rounds on acute inpatient medical wards: a descriptive study. BMJ Qual Saf.

[6] Hoffmann TC, Légaré F, Simmons MB, McNamara K, McCaffery K, Trevena LJ, Hudson GPP, Del Mar CB (2014). Shared decision making: what do clinicians need to know and why should they bother?. Med J Aust.

[7] Benecke M, Kasper J, Heesen C, Schäffler N, Reissmann DR (2020). Patient autonomy in dentistry: demonstrating the role for shared decision making. BMC Med Inform Decis Mak.

[8] Raab EL (2004). The parameters of informed consent. Trans Am Ophthalmol Soc.

[9] Brock DW (1991). The ideal of shared decision making between physicians and patients. Kennedy Inst of Ethics J.

[10] Bomhof-Roordink H, Gärtner FR, Stiggelbout AM, Pieterse AH (2019). Key components of shared decision making models: a systematic review. BMJ Open.

[11] Stiggelbout AM, Pieterse AH, De Haes JC (2015). Shared decision making: concepts, evidence, and practice. Patient Educ Couns.

[12] Allen M (2020). The value of values: shared decision-making in person-centered, value-based oral health care. J Public Health Dent Suppl.

[13] Neuman HB, Charlson ME, Temple LK (2007). Is there a role for decision aids in cancer-related decisions?. Crit Rev Oncol Hematol.

[14] LeRouge C, Nguyen AM, Bowen DJ (2022). Patient decision aid selection for shared decision making: a multicase qualitative study. Med Care Res Rev.

[15] Stacey D, Légaré F, Lewis K, Barry MJ, Bennett CL, Eden KB, Holmes-Rovner M, Llewellyn-Thomas H, Lyddiatt A, Thomson R, Trevena L (2017). Decision aids for people facing health treatment or screening decisions. Cochrane Database Syst Rev.

[16] Kalsi JS, Hemmings K (2013) The influence of patients’ decisions on treatment planning in restorative dentistry. Dent Update 40:698–700, 702–704, 707–708, 710. 10.12968/denu.2013.40.9.698 10.12968/denu.2013.40.9.698 24386761

[17] Touati R, Sailer I, Marchand L, Ducret M, Strasding M (2022). Communication tools and patient satisfaction: a scoping review. J Esthet Restor Dent.

[18] Al-Moghrabi D, Barber S, Fleming PS (2021). Removable retention: enhancing adherence and the remit of shared decision-making. Br Dent J.

[19] Drug and Therapeutics Bulletin (2013). An introduction to patient decision aids. BMJ.

[20] Marshman Z, Eddaiki A, Bekker HL, Benson PE (2016). Development and evaluation of a patient decision aid for young people and parents considering fixed orthodontic appliances. J Orthod.

[21] Motamedi-Azari F, Ryan FS, Jones E, Cunningham SJ (2020). A cross-sectional study investigating patients’ preferences regarding shared decision-making in adult orthodontic patients. Br Dent J.

[22] Coachman C, Calamita MA, Sesma N (2017). Dynamic documentation of the smile and the 2D/3D digital smile design process. Int J Periodontics Restorative Dent.

[23] Stacey D, Bennett CL, Barry MJ, Col NF, Eden KB, Holmes-Rovner M, Llewellyn-Thomas H, Lyddiatt A, Légaré F, Thomson R (2011). Decision aids for people facing health treatment or screening decisions. Cochrane Database Syst Rev.

[24] Veríssimo AH, Ribeiro AKC, de Medeiros AKB, de Melo LA, da Fonte Porto Carreiro A,  (2022). Factors associated with edentulous patients’ willingness about implant-supported complete denture: a multivariate analysis. Clin Oral Investig.

[25] Li X, Yang D, Meng M, Zhao J, Yin Y, Wang H, Zhang X, Liu Q, Li M, Liu J, Hao Y (2023). Shared decision-making in healthcare in mainland China: a scoping review. Front Public Health.

[26] Anagnostou A (2023). Shared decision making in food allergy: navigating an exciting era. Ann Allergy Asthma Immunol.

[27] Richter R, Jansen J, Bongaerts I, Damman O, Rademakers J, van der Weijden T (2023). Communication of benefits and harms in shared decision making with patients with limited health literacy: a systematic review of risk communication strategies. Patient Educ Couns.

[28] Oprea N, Ardito V, Ciani O (2023). Implementing shared decision-making interventions in breast cancer clinical practice: a scoping review. BMC MED Inform Decis Mak.

